# Low Tuberculosis (TB) Case Detection: A Health Facility-Based Study of Possible Obstacles in Kaffa Zone, Southwest District of Ethiopia

**DOI:** 10.1155/2020/7029458

**Published:** 2020-05-15

**Authors:** Mengistu Abayneh, Shewangizaw HaileMariam, Abyot Asres

**Affiliations:** ^1^School of Medical Laboratory Sciences, Mizan-Tepi University, Mizan-Aman, Ethiopia; ^2^Schools of Midwifery, Mizan-Tepi University, Mizan-Aman, Ethiopia; ^3^Departments of Public Health, Mizan-Tepi University, P.O. Box 260, Mizan-Aman, Ethiopia

## Abstract

**Background:**

In Ethiopia, the national TB case detection rate is becoming improved; still some districts are not able to meet their case detection targets which leads to ongoing spread of TB infections to family members and communities. This study was intended to assess possible obstacles contributing to low TB case detection in Kaffa zone, Southwest Ethiopia.

**Methods:**

A cross-sectional descriptive study involving qualitative and quantitative data was conducted from Mar. to Sep. 2019. Sociodemographic characteristics and data on duration of cough, whether sputum smear microscopy was requested or not, and data on TB knowledge and health care-seeking practice were collected from outpatients. Health care delivery barrier for TB case detection was also explored by using in-depth interview and FGD of health staff.

**Results:**

From 802 outpatients with coughing for 2 or more weeks of duration, 334 (41.6%) of them were not requested to have TB microscopic diagnosis. Of these, 11/324 (3.4%) of them were positive for TB after sputum smear microscopy. Only 24.2% of the outpatients were aware as they have had health education on TB disease. Twenty-eight percent of patients perceived that TB was due to exposure to cold air, and 13.5% could not mention any sign or symptom of TB. Amazingly, 54.2% of them did not have any information as current TB diagnosis and treatment is free. Thirty-five percent of the patients were taking antibiotics before visiting the health facility. The interrupted supply of TB diagnostic reagents, frequent electricity interruption, shortage of trained TB care providers, weak health information system, and weak active case finding practice were explored as the factors contributing to low TB case detection.

**Conclusion:**

Interrupted functioning of diagnostic centers, shortage of trained care providers, limited active TB case finding practice, weak health information system, and inadequate knowledge and health care-seeking practice of the patients were identified as contributors for low TB case detection. Thus, improving functioning of diagnostic centers, active TB case finding activities, and expanding health education on TB disease will help to improve TB case detection in the districts.

## 1. Background

Tuberculosis (TB) remains a major global health problem. Pulmonary tuberculosis (infection of the lungs) is the most common form of tuberculosis, as well as the most infectious, as transmission occurs from person-to-person via inhalation of respiratory droplets expelled when coughing or sneezing. About 1.7 billion people, 23% of the world's population, are estimated to have a latent TB infection and are thus at risk of developing active TB disease during their lifetime. The probability of developing TB disease is much higher among people infected with HIV; it is also higher among people affected by risk factors such as undernutrition, diabetes, smoking, and alcohol consumption [1, 2].

Globally, the best estimate is that 10.0 million people (range, 9.0–11.1 million) developed TB disease in 2017 [3]. The severity of national TB epidemics varies widely among countries. Africa and Asia are most heavily affected. In fact, 80% of all TB cases worldwide are from 22 high-burden countries, and in the majority of these countries, the TB control relies on passive case finding among individuals self-presenting to health care facilities followed by either diagnosis based on clinical symptoms or laboratory diagnosis using sputum smear microscopy [4, 5].

According to the WHO's goal, national TB control programmes around the world must reach at least 70% new, smear-positive TB cases (case detection), and 85% treatment success [6]. The targets for 2030 are an 80% reduction in the TB incidence rate and a 90% reduction in TB deaths as compared with levels in 2015 [3]. These targets cover short-term process targets related to the implementation of directly observed therapy (DOT) as well as epidemiological impact targets. However, different factors have been contributed for not all new TB cases are diagnosed and notified to the national TB programme, especially resource-limited settings [7, 8]. For instance, in the year 2017, 6.4 million new cases of TB that represented only 64% of the estimated 10.0 million new cases were officially notified to national authorities and then reported to the WHO worldwide [3]. The gaps between the estimated number of new cases and the number actually reported are due to a mixture of underreporting of detected cases and under diagnosis (either people do not access health care or because they are not diagnosed when they do) [3].

In the context of global TB, a major barrier to progressing towards TB control in Ethiopia and other high-burden countries is the TB “case detection rate,” which was stagnated at around 60% [9], meaning that estimated 80,000 Ethiopians who developed TB in 2014 were never diagnosed or treated. In Ethiopia, still, 55, 275 TB cases are not diagnosed or not notified, and 29,000 TB deaths were reported in 2017 [10]. The low TB case detection leads to increased ongoing spread of TB to family members and communities as each active case has capacity to infect 10–15 people per year [11]. Early case detection and prompt treatment cure the patients, break the transmission, and improve the TB control program.

In Ethiopia, the implementation and expansion of the DOTS strategy was supported by the expansion of health post, which is the lowest level of health facility and staffed by health extensions workers (HEWs) to play an important role in identifying and referring TB suspects to the next level of health care, that is, health centers for diagnosis and initiation of treatment [12–14]. However, most health facility workers malpractice the recommended TB infection control strategies knowingly or unknowingly. In addition, limitations of the TB diagnostic center, irregular supply of TB diagnosis and treatments, absence of active case finding practices might have direct impact on TB prevention and control. Therefore, researching these obstacles can have a central role in supporting guideline development for effectively preventing, diagnosing, and treating of TB infections by providing the evidence data needed. Thus, this study was intended to assess the existing obstacles that contributed to low TB case detection in Kaffa zone, Southwest Ethiopia.

## 2. Methods

### 2.1. Study Area and Period

A study was conducted in Kaffa zone, Southern Nations, Nationalities, and People's Region (SNNPR), which is located in Southwest Ethiopia from March to September, 2019. The city of the zone is Bonga town which is located at 468 kilometers from Addis Ababa, the capital city of Ethiopia. Administratively, the zone has 12 districts and two town administrations. This zone has one general public hospital, two primary hospitals, and 45 public health centers. Agriculture is the main source of livelihood for the community.

### 2.2. Study Design and Study Subjects

A health facility-based cross-sectional descriptive study involving qualitative and quantitative data was conducted to explore possible factors contributing to low TB case detection in Kaffa zone health facilities, Southwest Ethiopia. Three hospitals and seven health centers were selected purposively based on the availability of laboratory diagnosis services. To obtain quantitative data, consecutive patients with cough symptom for 2 or more weeks of duration were interviewed at their OPD exit. All patients with age greater than or equal to 18 years were first asked whether they have had coughing for 2 or more weeks of duration to be included in this study. Those patients with age groups less than 18 years and those critically ill were excluded. To obtain qualitative data, in-depth interview and focus group discussion (FGD) were conducted with health staff and technical staff working on the TB programme in the selected health facilities in the districts. The required sample size for outpatient attendants was determined using the single population proportion formula by assuming that the level of confidence is 95%, 5% margin of error, and P is the proportion of patients missing sputum smear microscopy, despite they present with cough symptoms for 2 or more weeks of duration.

### 2.3. Quantitative Data Collection

The quantitative data were obtained by face-to-face interviewing of outpatients at their OPD exit. Consecutive patients with coughing for 2 or more weeks of duration were interviewed to obtain data on duration of coughing, age, sex, area of residence, contact history, knowledge on symptoms and transmission of TB infection and the current cost of TB diagnosis and treatments, curability of TB disease, and first action taken at their first symptoms of cough which were collected using structured questionnaires. After the exit interview, each patient was checked whether the care provider attended laboratory investigation for sputum smear examination or not from their laboratory request and from the patient's record chart. Patients who were eligible for sputum smear examination but not be requested were advised to give sputum samples for laboratory examinations. Those patients with sputum smear-positive were traced and put on treatment.

### 2.4. Qualitative Data Collection

Qualitative data were collected with in-depth interview and FGD of health staff to explore TB health care service barriers. From each health facility, at least one head of health facilities, one laboratory head/staff, one OPD worker, and one pharmacist were interviewed. In addition, TB control program coordinators were interviewed from each district/woreda's health office. Subsequently, one FGD was conducted with a total of 11 health workers. Participants for the FGD were selected with the assistance of TB control program coordinators at districts/woredas and heads of health facilities. Accordingly, three laboratory technologists, two pharmacy professionals, two health officers, and four nurses were recruited for the FGD. Variables such as availability of continuous functioning of the TB diagnostic center, limitations of trained human resources for TB care delivery, shortage and irregular supply of TB diagnostic reagents and equipment, monthly review of the outpatient and laboratory registers to monitor progress of TB case detection and management, encouraging health extension workers and cured TB patients to help in educating and propagating the fact that TB is preventable and curable, community health education or social mobilization on TB and availability of active case finding programs, community-based screening programs, and household contact investigation were assessed using open-ended questionnaires and FGD.

### 2.5. Data Analysis

The data were first checked manually for their completeness and then entered into EpiData version 3.0 using double entry for verification. Descriptive statistics, including count and percentage, were calculated by using SPSS version 16 to describe the demographic characteristics and knowledge as well as health care-seeking practices of the subjects. Qualitative data were analyzed content wise and presented as thematically summarized quotes of participants as appropriate.

## 3. Results

### 3.1. Quantitative Findings

#### 3.1.1. Sociodemographic Characteristics of Study Participants

In this study, 802 patients with coughing for 2 or more weeks of duration were included. This number consisted of 408 (50.8%) males, and the rest of them were females. With regard to residence, 483 (60.2%) came from the rural parts of the district. About half, 416 (51.9%), of the patients were grouped under the age group of 18–34 years, and 291 (36.3%) of the respondents had no formal education, and 453 (56.5%) of them were engaged in farming. About 429 (53.5%) of them were married, and the rest of them were single (Table 1).

#### 3.1.2. Knowledge of Patients on TB Infections as a Possible Obstacle for Low TB Case Detection

With regard to knowledge of study participants, 393 (49.0%) of them responded that TB is transmitted from person-to-person, whereas 221 (27.6%) of them responded that TB comes from exposure to cold air. One hundred and fifty-seven (19.6%) of the respondents did not know any mode of transmission of TB infection. More than half (58.0%) of them were mentioning cough as a symptom of TB disease, and 108 (13.5%) of them could not mention any sign or symptom of TB disease. A small number, 172 (21.4%), of the respondents were also able to indicate that cough and weight loss as symptoms of TB disease. A significant number, 642 (80.0%), of the patients indicated that TB is curable, and 93 (11.6%) of them could not know whether TB is treatable or not. Similarly, 435 (54.2%) of them did not have any information as current TB diagnosis and treatment is free (Table 2).

#### 3.1.3. Health Care-Seeking Practice of Patients as a Possible Obstacle for Low TB Case Detection

Out of 802 patients with coughing for 2 or more weeks of duration, 283 (35.3%) of them were taking antibiotics, and 113 (14.0%) of them were practicing traditional medicine before coming to the health facility. Moreover, 262 (32.7%) of them did nothing before coming here. In this study, 145 (18.1%) patients have had previous contact with TB patients, and only 35 (24.1%) of them showed willingness to check their TB status. Only 190 (24.2%) patients were aware as they have had health education on TB infections (Table 3).

#### 3.1.4. Missing Sputum Smear Microscopy as a Possible Obstacle for Low TB Case Detection

In this study, from 802 patients with coughing for 2 or more weeks of duration, 334 (41.6%) were not requested to have TB microscopic diagnosis. Of these, 324 of them showed willingness to check their TB status, and 11 (3.4%) of them were positive for TB after sputum smear microscopy (Table 3).

### 3.2. Qualitative Findings

In this study, the possible obstacles for low TB suspect screening, low AFB microscopic uptake, and poor documentation and reporting were explored by qualitative methods. Variables such as shortage and irregular supply of laboratory reagents and equipment, inadequate trained human resources, regular interruptions of electric power, and poor health information system, including poor documentation, notification, and referral routines, poor health care service with limited outreach practices (poor health education on TB and poor active case finding), and poor diagnostic quality were the major themes of analysis. Each variable was narrated with expressive quotes of participants and presented thematically as follows.

#### 3.2.1. Shortage of Laboratory Reagents and Equipment

Respondents at health centers and peripheral hospital explained that the shortage and irregular supply of AFB and auramine-staining reagents, continuous interruption of electric power, and shortage of trained TB care providers with frequent turnover were identified as a significant problem of low TB diagnosis.

Respondents said that, “Although, auramine staining technique is more advanced than Ziehl–Neelsen technique; there was shortage of quality microscope (LED microscope) and Auramine stain especially in health centers. Even though we have one microscope for auramine staining technique, we faced frequent shortage of reagent to give continuous services. These may contribute for poor laboratory service which may contribute for low TB case detection in the region.”

Heads of health facilities and TB program manager at the district health office have also expressed their concern regarding inefficient resource utilization, especially at health facilities. Thus, as they have described, shortage of the resource coupled with unwise utilization continued to be interruptions for TB diagnosis services that needs great attention.

Respondents said that, “Poor and inefficient utilization of laboratory supply can be the main reason for frequent interruptions of the services. For example sometimes they didn't use pipettes to stain sputum smear slides instead they flood directly with containers.”

#### 3.2.2. Electric Power-Related Factors

Frequent interruptions of electric power and absence of alternative sources were a big problem for routine laboratory service. Therefore, this may lead the clinician to adapt treatment of the patients without laboratory request which may contribute to low TB case detection.

Respondents said that, “During interruptions of electric power, TB suspects advice and referred to nearby health facilities, but they prefer to go private clinics where there is no microscopic FAB service.”

#### 3.2.3. Laboratory Quality-Related Factors

Internal quality control practice was weak in all health facilities. Some laboratory technicians explained that they did not receive quality control training on microscopic sputum smear examination. In addition, there was no continuous supervision to explore the gaps with regard to TB diagnosis and treatment challenges.

Respondents said that, “Sometimes TB suspected patients may be reported as TB negative and become positive after requested for the second time. This indicates that TB positive patient can get additional time to spread the bacilli to many others. Moreover, Physicians and other health staffs may lose their confident on TB diagnostic laboratory and they may start to diagnose clinically.”

#### 3.2.4. Logistics Information System-Related Obstacles

According to respondent's expression, there was poor logistic information regulation in all health facilities. Respondents expressed that, “*It is the not the responsibility of laboratory technicians, rather it is pharmacy technicians to purchase, keep and control whole medical supplies including laboratory consumables. There is no internal consumption reporting system within the facility. Laboratory technicians request the laboratory consumables from pharmacy store as they needed. Due to this the pharmacy store keepers are not well scheduled to realize the types and time of reagent/supply going to be stocked out.”*

Respondents said that, “There is no proper transportation system of AFB reagents. Sometimes, from zonal store, AFB reagents were distributed to health facilities with light transparent containers that may reduce the quality of reagents.”

#### 3.2.5. Health Workers-Related Obstacles

Participants claimed that shortage of health workers coupled with high turnover might be attributed to poor health service quality in general and low TB case detection particularly. Respondents said that, *“There is high turnover and only few number of health workers in this health centers. Therefore, in my opinion these shortages of health work force, especially laboratory technicians can be one of the great challenges for low TB case detection. For example, there is only one laboratory technician in my health center and if he/she is in sick leave, the laboratory service is interrupted. Therefore, AFB microscopic service is interrupted which can be one of the great challenges for low TB case detection.”*

Health workers' attitude was also expressed as a problem of health care services. Health workers have a great role in accessibility, provision, and attainment of anticipated objectives of health services, but negligence and low commitments reverse the results.

Respondents said that, “Sometimes health workers neglect screening TB suspects and they treat patients with different antibiotics rather requesting AFB examination; even patients present with TB suggestive sign. In addition, non experienced laboratory technicians would not orient patients about the type and amount of sputum specimen. Therefore, sometimes patients bring saliva instead of sputum, and/or bring inadequate sputum, consequently resulting in incorrect laboratory diagnosis.”

#### 3.2.6. Health Information-Related Obstacles

Heads of health facilities and TB program coordinators have also expressed about poor recordkeeping and reporting system of health workers. In addition, there is no active TB case finding practice which was carried out by doing contact tracing that begins from facility records and regular review of registers. This was also may be due to negligence of care providers and sometimes overload of the work.

Respondents said that, “There is poor recording system and consequently there is poor health information use in some health facilities. Therefore, gaps of recording by itself might cause under reporting of diagnosed TB cases and consequently low TB case detection.”

Respondents said that, “There is no active case finding practice was carried out by doing contact tracing that begins from facility records. There is no strong referral linkage with health extension workers as they refer those patients with cough by finding them in the community.”

## 4. Discussion

Despite significant progress in diagnosis and treatment of tuberculosis (TB) over the past twenty years, millions of patients go undiagnosed or unreported every year. There are several possible reasons for low TB case detection rate and delayed treatment which were mentioned, including poor understanding of TB and its symptoms in the general population; poor knowledge about where to seek care; poor health service infrastructure with limited outreach; access barriers; poor diagnostic quality; limited human resources for health; poor TB knowledge among health providers; poor coordination of health services; and poor information systems, including notification and referral routines [4–7]. This study was conducted to explore existing obstacles for low TB case detection from health care delivery barriers and patients' perspectives in Kaffa zone, Southwest Ethiopia. Accordingly, this study showed that 334/802 (41.6%) patients with coughing for 2 or more weeks of duration were not requested to have TB microscopic diagnosis. This observation is lower than which was reported from South Nkwanta district of Ghana, in which only 25% of outpatients with persistent cough of 2 weeks and more duration had sputum examination done [15]. However, it is higher than the previous study finding in Kersa district, Southwest Ethiopia, in which 35.2% of tuberculosis suspects were not requested for microscopic examination of sputum smear [16]. In addition, our finding is higher than other study findings in Ethiopia, in which nearly 39% of estimated TB cases were missed: either not diagnosed, treated, or reported to the national tuberculosis program nonetheless improved health service coverage [14].

Actively asking all outpatients in primary health care facilities and hospitals for cough and referring them for sputum TB diagnostic test can yield substantial additional number of cases. In this study, of those patients not requested to have TB microscopic diagnosis, 11 (3.4%) of them were positive for TB after sputum smear microscopy. The finding of positive TB cases was relatively lower than which was reported from Gondar, Ethiopia, and India, in which the prevalence of undiagnosed pulmonary TB cases was 4.4% and 10.1%, respectively [17, 18]. Therefore, there is a possibility of these undiagnosed TB cases to pose a risk for the transmission of the disease, particularly among family members. Ensuring of comprehensive implementation of existing TB diagnostic algorithm requires that all staff in all parts of the health system are alert and know how to ask patients about TB symptoms and refer for TB diagnostic test as per guidelines.

The other fact is that successful TB infection control requires widespread of knowledge in the community around the signs and symptoms of TB and ways to control and treat it. In this study, only less than one-fourth (24.2%) of patients were aware as they have had health education on TB disease. Thus, less than half of the patients perceived as TB is transmitted from person-to-person, and a significant number of them still believe TB comes from exposure to cold air. In addition, even more than half (58.0%) of the patients responded cough as a symptom of TB infection, and still, quarter (13.5%) of them did not know any kind of symptoms. This finding is correlated with previous reports, in which a significant number of patients have gaps in the mode of transmission and symptoms of TB infection [19–21].

Amazingly, more than half (54.2%) of the respondents did not have any information as current TB diagnosis and treatment is free. In addition, a marked number (11.6%) of patients could not know whether TB disease is treatable or not. Similar finding was also reported in previous studies in Ethiopia, in which a significant number of patients have no information on curability and cost of diagnosis and treatment of TB disease [19–21]. The reported basic knowledge of patients about the symptoms and transmission and cost of diagnosis and treatment of TB has an important implication for the TB control program in the current study area in particular and in the country in general in that it could reduce diagnosis and treatment delay.

In this study, the common pattern of care-seeking practice among the patients first was self-treatment. That is, a significant number of the patients (35.3%) responded as they attempted taking antibiotics from the pharmacy for initial cough symptoms (Table 3). When symptoms worsened (coughing with blood), the patients found their own way to health facilities, and they may have an opportunity to be screened for TB infection. This is in accordance with earlier studies in different parts of Ethiopia [21, 22], in which a significant number of the respondents have gaps on TB knowledge and first practiced self-treatment before coming to the health facilities. Therefore, patients' knowledge regarding TB infection may have a negative impact on patients' attitude towards health care-seeking behavior and preventive methods as most people with such beliefs may not visit health facilities or they may consider various traditional alternatives.

Another possible factor that could be contributing to low TB case detection rate in the districts may be related with TB care-providing activity of health facilities. The qualitative findings of this study showed that continuous functioning of diagnostic centers, especially in health centers, was found to be inadequate since many laboratories interrupt their sputum smear examination due to shortage of reagents, inadequate trained human resources, and interruptions of electric power. The laboratory heads of many health centers described that even though auramine staining technique is more advanced than Ziehl–Neelsen technique, there was shortage of auramine “A” stain and quality microscope (fluorescence microscope) in health centers. In addition, an interruption of electric power was very problematic. These might be contributed to inadequate laboratory service which may contribute to low TB case detection in the region. Similar challenges were also reported from different resource-limited countries, which had been resulted in low TB case detection [14–16, 23, 24].

In addition, participants explained that there was significant shortage of trained human power with high turnover, especially in health centers. There are few opportunities for the training of staff and little staff capacity to handle high-volume workloads. Participants also expressed that relatively low motivation of care providers was observed at service delivery points. Sometimes, health workers neglect screening TB suspects as per national guidelines. They treat patients with different antibiotics rather requesting for AFB examination; even patients present with TB suggestive sign. Similar finding has also been reported from other parts of Ethiopia and from other reviews [16, 23, 24]. If there are trained and adequate health workers, then the service quality can get better, and the reverse is true for inadequate and untrained human resources.

Moreover, some laboratory technicians did not receive quality control training on microscopic sputum smear examination and would not orient patients properly about the type and amount of the sputum specimen that the patient should bring. Therefore, sometimes, patients bring saliva instead of sputum and/or bring inadequate sputum, consequently resulting in incorrect laboratory diagnosis. This indicates that the TB-positive patient can get additional time to spread the Bacilli to many others. Moreover, physicians and other health staff may lose their confidence on the TB diagnostic laboratory, and they may start to diagnose clinically. This finding is also supported by different studies presented in resource-limited countries [23, 25–27]. Thus, shortage of well-trained health care providers coupled with low commitment contributed to inadequate service quality in general, which can affect TB case detection in particular.

Well-furnished and adequate working spaces and electric power are some of the basic requirements in the smear microscopy laboratory [27]. In contrast, in our finding, many laboratories, spatially in health centers, have inadequate work space (only one room for all laboratory activities) and faced frequent interruptions of electric power. Similar challenges were also reported by the study finding in resource-limited countries, in which many smear microscopy laboratories are single-room and understaffed with poorly maintained microscopes, and some of these laboratories lack consistent sources of electricity [11, 23, 27].

Health information is essential for evidence-based planning and need-based allocation of the health resource [28, 29]. To be ensured of these, health workers and other staff should have complete and accurate recordkeeping systems at health facilities and submitted to respective bodies in timely basis. However, most respondents expressed that there was still a significant gap of recording, reporting, and utilization of logistic information systems at health facility level. Previous studies in other parts of Ethiopia also concluded that there were gaps in TB data documentation systems [16, 23, 24, 26]. Therefore, gaps of recording and reporting by itself might cause underreporting of diagnosed TB cases and consequently low TB case detection.

In high-incidence countries, TB control relies on the passive case finding among individuals self-presenting to health care facilities followed by either diagnosis based on clinical symptoms or laboratory diagnosis using sputum smear microscopy [5, 25]. However, recent WHO guidelines recommended that active case finding, contact investigations, and using fluorescent (LED) microscopy and Gene Xpert MTB/RIF assay can help to improve TB case detection [7, 30]. A study in India concluded that household contact investigation could attribute to additional 63% TB cases as compared to passive case detection alone [18]. In Ethiopia also, health extension workers (HEWs) and project supervisors were trained on methods to systematically identify people with suspected TB in the community. However, in this study, respondents expressed that there is interrupted/weak contact tracing and active case finding practice which was carried out at many health facilities. In addition, with frequent shortage of auramine stain, only a few health facilities were using advanced fluorescent (LED) microscopy, and one Gene Xpert MTB/RIF assay was available only at Bonga general hospital. Thus, increasing access to advanced TB diagnostic tools and strengthening involvement of HEWs in expanding health education on TB infection as they participate in active case finding may have an impact in improving TB case detection and raising communities' awareness towards TB infection.

### 4.1. Limitations of the Study

The study was health facility-based; therefore, we missed people with TB symptoms but not have attended health facilities. Since the variables studied were perceived as obstacles for low TB case detection, authors performed descriptive analysis instead of significant tests.

## 5. Conclusion

In conclusion, inadequate functioning of diagnostic centers due to inadequate supply of reagents and equipment, interruptions of electric power and shortage of well-trained health workers, and low level of active TB case finding activities by health staff, weak record review systems, and gaps of TB knowledge and health care-seeking practice of the patients were identified as possible contributors for low TB case detection rate in the districts. Therefore, improving the functions of TB diagnostic centers and active TB case finding activities and expanding health education on TB sign and symptoms will go a long way to improve TB case detection in the districts. In addition, a referral network between community providers and TB diagnostic facilities should be established to reduce diagnostic delays.

## Figures and Tables

**Table 1:** Sociodemographic characteristics of patients who were coughing for 2 or more weeks of duration in Kaffa zone health facilities, Southwest district of Ethiopia, 2019.

Variables	Patients with cough (*n* = 802)		
	Categories	Number	%
Age	18–34	416	51.9
	35–54	261	32.5
	≥55 years	125	15.6

Sex	Male	408	50.8
	Female	394	49.1

Marital status	Married	429	53.5
	Single	373	46.5

Residence	Urban	319	39.8
	Rural	483	60.2

Educational background	Illiterate	291	36.3
	Elementary	399	49.8
	Secondary +	112	14.0

Occupations	Farmer	453	56.5
	Employee	103	12.8
	Merchant	56	7.0
	Daily laborer	57	7.1
	Student	133	16.6

**Table 2:** Knowledge of patients with coughing for 2 or more weeks of duration on TB disease in Kaffa zone health facilities, Southwest district of Ethiopia, 2019.

Variables	Patients with cough (*n* = 802)		
	Categories	Number	%
Mode of transmission	Person-to-person	393	49.0
	Exposure to cold air	221	27.6
	Sharing utensils	31	3.9
	Nothing known	157	19.6

Signs and symptoms of TB	Cough only	465	58.0
	Cough with weight loss	172	21.4
	Weight loss only	57	7.1
	Nothing known	108	13.5

Curability of TB	Curable	642	80.0
	Not curable	67	8.4
	Nothing known	93	11.6

Cost of TB diagnosis and treatment	Free	266	33.2
	Paid	101	12.6
	Not have information	435	54.2

**Table 3:** Health care-seeking practice of patients with coughing for 2 or more weeks of duration in Kaffa zone health facilities, Southwest district of Ethiopia, 2019.

Variables	Patients with cough (*n* = 802)		
	Categories	Number	%
Duration of cough	For ≤2 weeks	421	52.5
	For >2 weeks	381	47.5
Sputum smear microscopy requested	Requested	468	58.4
	Not requested (missed)	334	41.6
Willingness to check their TB status (from 334)	Yes	324	97.0
	No	10	3.0
Sputum smear microscopy results (from 324)	Positive	11	3.4
	Negative	313	96.6
The first action taken before coming here	Nothing done	262	32.7
	Visited health facilities	144	18.0
	Traditional treatment	113	14.0
	Taking antibiotics from pharmacies	283	35.3
TB contact history	Yes	145	18.1
	No	657	81.9
Informed TB status of their relatives	Health workers	48	33.1
	Relatives	97	66.9
Those checked TB status	Tested	35	24.1
	Not tested	110	75.9
Ever having health education on TB disease	Yes	190	24.2
	No	608	75.8

## Data Availability

All the data supporting our findings are incorporated within the manuscript.

## Abbreviations

TB: Tuberculosis
OPD: Outpatient department
FGD: Focus group discussion
HEW: Health extension worker
DOT: Directly observed treatment
